# Switching from Neutral Protamine Hagedorn Insulin to Insulin Glargine 300 U/mL Improves Glycaemic Control and Reduces Hypoglycaemia Risk: Results of a Multicentre, Prospective, Observational Study

**DOI:** 10.1155/2020/8751348

**Published:** 2020-04-08

**Authors:** B. Wolnik, D. Wiza, T. Szczepanik, A. Syta, T. Klupa

**Affiliations:** ^1^Medical University of Gdansk, Department of Hypertension and Diabetology, Gdansk, Poland; ^2^Poznan University of Medical Sciences, Department of Internal Medicine and Diabetology, Poznan, Poland; ^3^Starkiewicz Hospital, Zaglebie Oncology Center, Dabrowa Gornicza, Poland; ^4^Sanofi-Aventis Poland, Medical Affairs, Warszawa, Poland; ^5^Jagiellonian University Medical College, Department of Metabolic Diseases, Kraków, Poland

## Abstract

Type 2 diabetes mellitus (T2DM) is a major cause of morbidity and mortality worldwide and is an important public health issue. A significant proportion of insulin-treated patients with T2DM do not reach target glycated haemoglobin (HbA1c) values, which ultimately increases their risk of long-term microvascular and macrovascular complications. One potential option to improve diabetes control in these patients may be the use of new insulin formulations including second-generation basal insulin analogues such as insulin glargine 300 U/mL (Gla-300). Several published randomised controlled trials have assessed the clinical effectiveness of Gla-300, mostly versus insulin glargine 100 U/mL as well as insulin degludec. However, there is limited information about the real-world effectiveness of Gla-300 when patients are transitioned directly from neutral protamine Hagedorn (NPH) human basal insulin. The primary objective of this study was to evaluate the effectiveness of Gla-300, defined as the percentage of participants with an HbA1c reduction of ≥0.5%, 6 months after switching from NPH insulin, in participants with T2DM. Secondary objectives included the safety assessment based on the percentage of patients experiencing ≥1 episodes and the number of hypoglycaemic episodes by category: severe, symptomatic, symptomatic confirmed, diurnal or nocturnal, change in body weight, and insulin dose. A total of 469 participants completed the 6-month observation period. Mean baseline HbA1c was 9.19%. The percentage of participants with a ≥0.5% improvement in HbA1c from baseline was 71.7% at 6 months. Mean HbA1c decreased at 3 and 6 months by 0.77% (±0.98) and 1.01% (±1.12), respectively (*p* < 0.00001 versus baseline), while fasting glycaemia decreased by 32 mg/dL and 37 mg/dL, respectively (*p* < 0.00001 versus baseline). There were moderate increases in the doses of both Gla-300 and, if used, short-acting insulins during the 6 months of observation. The percentage of participants with ≥1 hypoglycaemia event during the preceding 4 weeks decreased significantly from baseline to 3 and 6 months, as did the proportion with symptomatic hypoglycaemia at night (*p* < 0.00001 versus baseline). No participants had severe hypoglycaemia after a switch to Gla-300. Body mass, waist and hip circumferences, and waist : hip ratio did not change significantly. In conclusion, this large, prospective, observational study demonstrated that switching from NPH insulin to Gla-300 resulted in a significant improvement in HbA1c, with only a moderate increase in insulin dose, a decreased risk of hypoglycaemia, and no increase in body weight.

## 1. Introduction

Type 2 diabetes mellitus (T2DM) is a major cause of morbidity and mortality worldwide and a significant public health issue. Optimising blood glucose control, especially in insulin-treated patients, is challenging because it requires decreasing glycated haemoglobin (HbA1c) to be balanced against potentially increasing the risk of hypoglycaemia. Hypoglycaemia is considered to be the major barrier to achieving optimal control with insulin treatment of T2DM [1]. To avoid hypoglycaemia, insulin-treated patients with T2DM may intentionally maintain their plasma glucose levels above recommended values [2, 3]. A significant proportion of patients with T2DM do not reach target HbA1c [4–7], which ultimately increases their risk of long-term microvascular and macrovascular complications. Fear of hypoglycaemia is also considered to contribute to suboptimal glucose control [8].

One potential approach to improve HbA1c without increasing hypoglycaemia risk in insulin-treated patients with T2DM may be the use of novel, safer analogue insulin formulations. Switching from human neutral protamine Hagedorn (NPH) basal insulin to long-acting, first-generation basal insulin analogues significantly decreases the risk of hypoglycaemia in patients with T2DM [9]. In addition, the use of second-generation basal insulin analogues that have peakless pharmacokinetic profiles and longer duration of action was shown to further decrease hypoglycaemic risk [10, 11]. However, no studies have evaluated the real-world effectiveness of second-generation basal insulins in patients transitioned from NPH human basal insulin. Addressing this issue would be of high clinical importance since in many countries, including Poland, NPH insulin is still commonly used.

## 2. Study Aims

The primary objective of this study was to evaluate the effectiveness of insulin glargine 300 U/mL (Gla-300) in participants with T2DM previously treated with NPH insulin in Polish diabetes centres. Clinical effectiveness was defined as the percentage of participants with an HbA1c reduction of ≥0.5% 6 months after switching to Gla-300. Secondary objectives included assessment of change from baseline to months 3 and 6 in HbA1c, percentage of participants achieving individually defined HbA1c targets, fasting blood glucose, mean of seven-point self-monitoring of blood glucose (SMBG) profile, insulin dose, and body weight. Safety was also evaluated as a secondary endpoint according to the percentage of participants with ≥1 event and the number of hypoglycaemic events by category: symptomatic, confirmed (if blood glucose ≤ 3.9 mmol/L (70 mg/dL)), severe (i.e., requiring the assistance of others to administer carbohydrates or glucagon or apply other corrective actions), diurnal (wake time), and nocturnal (during sleep).

## 3. Material and Methods

### 3.1. Study Setting

Participating investigators were diabetologists, who routinely treat patients with T2DM at diabetes centres/diabetologist practices and who accepted the principles of the protocol. At each centre, consecutive patients meeting the eligibility criteria were enrolled, until the target accrual of 500 participants was reached.

This registry was conducted in accordance with the principles laid down by the 18th World Medical Assembly (Helsinki, 1964) including all subsequent amendments. Registry conduct also complied with all international guidelines and national laws and regulations of Poland, as well as any applicable guidelines. The sponsor (Sanofi-Aventis Poland) ensured that all necessary regulatory submissions (e.g., Institutional Review Board/Independent Ethics Committee approval) were performed in accordance with local regulations including those relating to data protection.

### 3.2. Eligibility

Eligible patients were aged ≥18 years, had T2DM, and were switched to Gla-300 from NPH insulin being the part of following treatment regimens: basal+oral antidiabetic agents (OAAs), basal/bolus (bolus with either regular human insulin or analogues), or human regular premixed (containing NPH as the basal component). Previous NPH insulin treatment should have lasted at least 6 months with basal, basal/bolus, or human regular premixed insulin regimens. Previous NPH insulin treatment should have been of at least 6 months' duration. Patients were also required to have a most recent HbA1c assessment from within 4 weeks before enrolment of ≥8% while on NPH insulin treatment. All patients gave consent to participate in this observational study. Exclusion criteria were type 1 diabetes, participation in another clinical study in parallel or within the past 3 months, allergy to insulin glargine, noncooperative patient (as judged by the investigator), current or previous drug or alcohol abuse within the previous 2 years, and pregnancy.

### 3.3. Assessments

Patient data were collected at baseline and at 3 and 6 months (±2 weeks) after enrolment. Demographic data included age, sex, body weight, height, blood pressure, body-mass index (BMI), waist and hip circumferences (used to calculate the waist : hip (W : H) ratio), education level, and place of residence. Data were also acquired on clinical characteristics, including completion of training in diabetes self-management, participation in regular physical exercise, intake of an individually adjusted diet, participation in SMBG, insulin regimen, history of T2DM that led to insulin prescription, previous treatment with OAAs and insulin before initiation of Gla-300 (including type of regimen: basal, basal/bolus, or insulin mixture), and current treatment prescribed after switch to insulin Gla-300, including use of short-acting insulin(s) and OAAs.

Analysed parameters included change from baseline in body weight and waist and hip circumference at 3 and 6 months (±2 weeks). In addition, changes in efficacy parameters were evaluated over the same time period, including HbA1c and fasting glycaemia (based on laboratory values or SMBG profile consisting of seven or fewer points, as provided by the participant). The number of episodes of symptomatic, symptomatic confirmed (≤70 mg/dL (≤3.9 mmol/L)), severe, diurnal, or nocturnal hypoglycaemia during the 4-week periods before Gla-300 initiation and the 3- and 6-month assessments was recorded. During the 6-month observation period, data were also collected on all adverse events (AEs) and serious adverse events (SAEs), including assessment of severity, relation to Gla-300 treatment, and outcome.

### 3.4. Statistical Analysis

Statistical analysis of the collected data was performed using descriptive measures according to a predefined statistical analysis plan. Continuous variables were described by patient numbers, mean ± SD, median, and range. For categorical variables, absolute and percentage of frequencies were determined. Paired, two-tailed Student's *t*-test was applied to test for differences in values between baseline and after 3 and 6 months. The chi-squared test was used for nonparametric variables.

## 4. Results

### 4.1. Patient Population

The study was conducted at 51 Polish diabetes centres and diabetologist practices (although three sites enrolled no participants). A total of 499 eligible patients were enrolled, of whom 469 completed 6 months of observation during Gla-300 treatment. Participant flow is presented in Table 1. The 30 participants who were not assessed at 3 and/or 6 months were excluded from the statistical analysis. The characteristics of the 469 participants who completed 6 months of follow-up are presented in Table 2.

### 4.2. HbA1c, Fasting Glycaemia, and Daily Blood Glucose Profile

In the primary endpoint analysis, 71.7% of participants achieved a decrease in HbA1c of ≥0.5% from baseline to 6 months after switching from NPH insulin to Gla-300. At 3 and 6 months, respectively, there were significant decreases in mean HbA1c of 0.77% (±0.98) and 1.01% (±1.12) (*p* < 0.00001 versus baseline) and fasting glycaemia of 32 mg/dL and 37 mg/dL (*p* < 0.00001 versus baseline). Furthermore, a consistent decrease in mean glycaemia values in the daily profile was noted during 6 months of observation (Table 3). As glycaemia profile data collection was not obligatory and was provided for only 44.8% of participants at different time points, these data were not analysed statistically.

### 4.3. Diabetes Treatment Adjustment during Observation

Before switching to Gla-300 due to uncontrolled HbA1c (≥8%), all enrolled patients were treated with NPH insulin (including NPH being the basal component of premixed formulation) with or without concomitant OAA administration. Prior to the switch, the mean dose of long-acting NPH insulin was 38 U (±22), the mean dose of short-acting insulin was 32 U (±20), and the mean total insulin dose was 61 U (±32). Metformin was administered concomitantly in 67.6% of participants (mean daily dose: 2370 ± 770 mg), whereas 13.2% received sulphonylureas. Less frequently used OAAs included sodium-glucose transport protein-2 inhibitors (6.6%), alpha-glucosidase inhibitors (2.3%), dipeptidyl peptidase-4 inhibitors (1.5%), thiazolidinediones (0.9%), and glucagon-like peptide-1 receptor agonists (0.2%).

**Figure 1:**
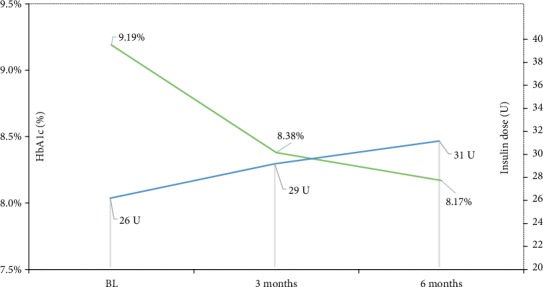
Relative changes in HbA1c versus Gla-300 dose in the study population. Blue line: glargine 300 doses at baseline, 3 months, and 6 months. Green line: HbA1c values at baseline, 3 months, and 6 months.

There was a moderate increase in the doses of both Gla-300 and short-acting insulins during the 6-month observation period, with an increase in the total insulin dose of 13% compared with baseline (Table 4). The relative decrease in HbA1c versus the increase in Gla-300 dose is summarised in Figure 1. Administration of OAAs slightly decreased during the study (Table 5).

### 4.4. Change in BMI and W/H Ratio

Body weight, waist and hip circumferences, and W/H ratio did not change significantly during observation (Table 6).

### 4.5. Hypoglycaemia

The number of participants with ≥1 diurnal or nocturnal hypoglycaemia episode during the previous 4 weeks decreased significantly from baseline at 3 and 6 months, as did the number with symptomatic hypoglycaemia at night (*p* < 0.00001 versus baseline) (Table 7). No severe hypoglycaemia was reported after a switch to Gla-300. There was a nonsignificant trend for a decrease in the rate of symptomatic confirmed hypoglycaemia at night versus baseline. The frequency of symptomatic and symptomatic confirmed hypoglycaemia at night was reduced at 6 months versus baseline by 43% and 18% (NS), respectively (Table 8).

### 4.6. Safety

Thirty-seven AEs were reported in 29 participants, of which 11 were SAEs. According to investigator assessment, 4 AEs were severe, 25 AEs were moderate, and 8 AEs were mild. One SAE resulted in death, due to acute circulatory insufficiency. Another participant died due to progression of cancer 4 months after enrolment. As this patient did not have an assessment at 3 months after inclusion, the patient was considered lost to follow-up and the event was not reported as an SAE. None of the reported AE was considered as related to Gla-300 administration.

## 5. Discussion

This study is aimed at assessing the real-world effectiveness of switching from NPH basal insulin to Gla-300 in patients with T2DM. To our knowledge, it is the first large, prospective observational study addressing this issue. We found that the switch from NPH insulin to Gla-300 resulted in a significant reduction in HbA1c of 1.01% at 6 months relative to baseline. This improved glycaemic control was achieved with only a moderate increase in the doses of both Gla-300 and short-acting insulins during the 6-month observation period, with an increase in total insulin dose of 13% versus baseline. Body mass, waist and hip circumferences, and W/H ratio did not change significantly. A crucial finding was that the number of participants with ≥1 hypoglycaemia episode decreased significantly at 3 and 6 months compared with the 4-week period before inclusion, as did the rate of symptomatic hypoglycaemia at night. Importantly, no severe hypoglycaemia was reported during the entire observation period.

One of the few prior studies reporting on a direct switch from NPH insulin to Gla-300 in at least a subset of participants was a prospective, observational, multicentre trial by Seufert et al. [12]. Patients were switched from their current basal insulin to Gla-300 due either to inadequate glycaemic control or occurrence of hypoglycaemia. Baseline basal insulins were insulin glargine 100 (53.0%), NPH insulin (21.9%), insulin detemir (13.7%), or others (%). In the primary endpoint analysis, 27% of patients achieved a fasting plasma glucose (FPG) target of <110 mg/dL after 12 months. Furthermore, 54% of patients achieved the combined endpoint of either FPG < 110 mg/dL or an individualised HbA1c target. The highest combined target achievement was attained by patients switching from insulin detemir (63.5%) or NPH insulin (59.0%). There was a reduced incidence of hypoglycaemia, especially nocturnal episodes, while only minor changes in weight occurred. The dose of Gla-300 increased from 0.30 U/kg/day at baseline to 0.36 U/kg/day at month 12.

Overall, Seufert et al. showed general trends similar to those of the present study, with a significant decrease in HbA1c, reduction in hypoglycaemia rates, no significant weight gain, and only a minor increase in insulin dose. The major difference, however, was that the former study focused on patients receiving basal insulin only (in combination with oral agents), whereas our study also included patients on basal and prandial insulins. In addition, the population enrolled by Seufert et al. was heterogenous with respect to oral agents and basal insulins used at baseline, with only a small proportion of patients receiving NPH insulin.

Similarly, Gupta et al. reported the effect of switching to insulin glargine 300 from other base insulins on clinical outcomes in patients with type 2 diabetes [13]. This was a retrospective observational study using medical record data obtained by a physician survey for part of which concerned switching to treatment with Gla-300 from treatment with another basal insulin. The daily dose of basal insulin was significantly lower after switching to treatment with Gla-300 from treatment with another basal insulin (0.73 U/kg vs. 0.58 U/kg). The mean haemoglobin A1c level was significantly lower after switching than before switching (-0.95 percentage points). Hypoglycemic events per patient-year were significantly lower (relative risk 0.17). Unfortunately the studied group was very heterogeneous with respect to ethnicity, mode of treatment, and basal insulin used. A comparison to our study does not make too much sense since patients switching to glargine 300 from NPH insulin were excluded from the analysis. In addition, the studied population was characterized with much higher BMI as compared to ours.

Hidvegi et al. reported a 6-month, prospective, multicentre, noninterventional, observational, single-arm study evaluating the effectiveness of Gla-300 when used as a part of basal/bolus therapy in patients with T2DM [14]. Patients switching from human insulin-based basal/bolus therapy to Gla-300-/insulin glulisine-based treatment were eligible. Compared with baseline, after 6 months of therapy, HbA1c decreased by 1.36%, with no weight gain, while total insulin dose increased moderately by 9%, which was entirely a result of Gla-300 uptitration. The mean number of hypoglycaemia events per patient per year decreased from 9.76 before the study by 2.3 from baseline to month 6. These data are broadly consistent with the present study. However, it should be emphasised that Hidvegi et al. included a population that was homogenous with regard to types of insulin and model of therapy used at baseline. In contrast, although all participants in our study received NPH insulin, it was used in different types of regimen and combined with different prandial insulins.

Other available data comparing Gla-300 vs. NPH insulin provide consistent results regarding hypoglycaemia risk but variable findings with respect to HbA1c. In a recent network meta-analysis, Freemantle et al. found that, compared with NPH insulin, Gla-300 provided a comparable change in HbA1c and body weight, with a significantly lower nocturnal hypoglycaemia rate (risk ratio: 0.18; 95% confidence interval: 0.05 to 0.55) [15]. The effect on hypoglycaemia risk was consistent with our findings. The lack of effect on HbA1c could be explained by the inclusion in the network meta-analysis of only randomised controlled trials, a majority of which followed a treat-to-target design. In contrast, the present study was based on the use of two sequential therapies.

It is difficult to directly compare our data with that of previous studies of the effectiveness and safety of Gla-300, since most prior reports have compared this agent with insulin glargine 100 U/mL or other insulin analogues. However, despite the differences in study designs, our study should be discussed in the context of selected previous analyses. For example, EDITION 1 was a multicentre, open-label, parallel-group study of patients with T2DM receiving current basal therapy with either insulin glargine 100 U/mL or NPH insulin, together with mealtime insulins [16]. Participants were randomised to receive once-daily injections of either Gla-300 or insulin glargine 100 U/mL. At the end of the observation period, HbA1c reduction was equivalent between regimens, but fewer participants reported one or more confirmed or severe nocturnal hypoglycaemic events between week 9 and month 6 with Gla-300. However, in contrast to our study, daily basal insulin dose in EDITION 1 increased from 0.67 U/kg/day to 0.97 U/kg/day after 6 months. Mealtime insulin doses increased slightly in the first 2 weeks but were unchanged from baseline thereafter. In addition, unlike the present study, EDITION 1 reported an increase in body weight of 0.9 kg in both treatment groups. This difference could be explained by the very rigid titration rules in EDITION 1, compared with the more flexible approach to titration adopted in real-world practice. However, glycaemic control at the end of our study (mean HbA1c: 8.17%) was far from optimal and even higher doses of Gla-300 would be required to meet therapeutic targets. Other factors potentially contributing to the differences in insulin dose and body weight outcomes between EDITION 1 and the present study are the far higher BMI (36.6 versus 32.5 kg/m^2^, respectively) and the much greater total daily insulin dose at baseline (126.3 versus 61.0 U, respectively).

Pettus et al. recently reported an interesting study that applied conventional and advanced analytical approaches to model, predict, and compare hypoglycaemia rates in patients with T2DM receiving Gla-300, or first-generation (insulin glargine 100 U/mL or insulin detemir) or second-generation (insulin degludec) basal insulin analogues, utilising a large, real-world database [17]. The analysis showed that rates of severe hypoglycaemia were approximately 50% lower with Gla-300 versus insulin glargine 100 U/mL or insulin detemir in insulin-naïve individuals, and 30% lower versus insulin detemir in patients switched from basal insulin. Like our study, this analysis showed a reduction in hypoglycaemia risk with Gla-300, but more detailed comparisons are precluded by differences in study design and the absence of patients on NPH insulin in the study by Pettus et al.

To summarise, while there is strong evidence that switching from NPH insulin to Gla-300 in patients with T2DM decreases hypoglycaemia risk without causing weight gain, data from randomised controlled trials are inconsistent regarding improvement in HbA1c. There is a lack of real-life, prospective, observational data comparing clinical effectiveness of NPH insulin vs. Gla-300, which is in part addressed by our study.

## 6. Conclusions

This large, prospective, observational study shows that a direct switch from NPH insulin to Gla-300 results in a significant improvement in diabetes control (as reflected in HbA1c) and a reduction of the number of patients experiencing ≥1 hypoglycaemia, without an increase in body weight, which comes at the cost of only a moderate increase in insulin dose. Gla-300 is a safe and effective therapeutic option in patients with T2DM who are uncontrolled on other insulins.

## Figures and Tables

**Table 1:** Participant flow.

Participants	*n*
Enrolled	499
Completed 6 months of observation	469
Discontinued (not assessed at 3 and/or 6 months)	30
Unknown reason for discontinuation	19
Incomplete data at baseline	13
Lost to follow-up	7
Switched to other insulin	2
Death	1
Inefficacy	1

**Table 2:** Participant characteristics.

Sex	
Men, *n* (%)	222 (47.3)
Women, *n* (%)	247 (52.7)
Age (years), mean±SD	64.8 ± 9.1
Height (cm), mean±SD	167 ± 9
Body weight (kg), mean±SD	90.8 ± 17.4
BMI (kg/m^2^), mean±SD	32.5 ± 5.7
W/H ratio, mean±SD	0.99 ± 0.09
Blood pressure (mm Hg), mean	139/80
Duration of T2DM (years), mean±SD	14.4 ± 7.7
Training in diabetes management since diagnosis^∗^, *n* (%)	
Individual	304 (65)
Group	84 (18)
Self-education	112 (24)
None	56 (12)
Individualised diabetes diet, *n* (%)	
Yes	178 (38)
No	291 (62)
Regular physical exercise (≥30 minutes, four times per week), *n* (%)	
Yes	80 (17)
No	389 (83)
SMBG, *n* (%)	
Yes	403 (86)
No	66 (14)
Place of residence, *n* (%)	
Voivodeship capital	113 (24
Other city	211 (45)
Village	145 (31)
Education level, *n* (%)	
University	56 (12)
High school	286 (61)
Elementary	127 (27)
Insulin NPH regimen, *n* (%)	
Basal	130 (28)
Basal-bolus	331 (70)
Human regular premixed insulin	8 (2)

^∗^Multiple options could be recorded. BMI: body-mass index; NPH: neutral protamine Hagedorn; SD: standard deviation; SMBG: self-monitoring of blood glucose; T2DM: type 2 diabetes mellitus; W/H: waist : hip ratio.

**Table 3:** HbA1c and glycaemia (fasting and daily profile measurements).

Parameter, mean (SD)	Baseline	Month 3	Month 6
HbA1c (%)	9.19 (1.11)	8.38 (1.12)^∗^	8.17 (1.17)^∗^
Glycaemia (mg/dL)			
Fasting	178 (46)	146 (38)^∗^	141 (41)^∗^
After breakfast	199 (55)	168 (44)	166 (42)
Before lunch	174 (54)	146 (43)	146 (45)
After lunch	210 (64)	183 (51)	178 (48)
After dinner	177 (51)	155 (51)	152 (46)
Before sleep	189 (57)	158 (49)	156 (45)

^∗^ *p* < 0.00001 versus baseline. SD: standard deviation.

**Table 4:** Gla-300, short-acting and total insulin doses during observation.

Insulin dose (U), mean (SD)	Baseline	Month 3	Month 6
Gla-300	26 (11)	29 (12)	31 (13)
Short-acting insulin (human regular or analogue)	44 (22)	47 (23)	48 (25)
Total	61 (32)	67 (34)	69 (35)

Gla-300: insulin glargine 300 U/mL; SD: standard deviation.

**Table 5:** Change in concomitant OAA administration during observation.

Patients, *n* (%)	Before switch	Baseline	Month 3	Month 6
Metformin	317 (67.6)	311 (66.3)	308 (65.7)	308 (65.7)
Metformin in combination	10 (2.1)	14 (3.0)	11 (2.3)	12 (2.6)
Sulphonylureas	62 (13.2)	50 (10.7)	49 (10.4)	44 (9.4)
Alpha-glucosidase inhibitors	13 (2.8)	11 (2.3)	9 (1.9)	10 (2.1)
DPP-4 inhibitors	12 (2.6)	7 (1.5)	4 (0.9)	4 (0.9)
SGLT-2 inhibitors	31 (6.6)	34 (7.2)	32 (6.8)	36 (7.7)
Thiazolidinediones	4 (0.9)	3 (0.6)	4 (0.9)	6 (1.3)
GLP-1 receptor agonists	1 (0.2)	1 (0.2)	1 (0.2)	0

DPP-4: dipeptidyl peptidase-4; GLP-1: glucagon-like peptide-1; OAA: oral antidiabetic agent; SGLT-2: sodium-glucose transport protein-2.

**Table 6:** Change in weight, BMI, waist and hip circumferences, and W/H ratio during observation.

Parameter, mean (SD)	Baseline	Month 3	Month 6
Body weight (kg)	90.8 (17.4)	90.7 (17.3)	90.5 (17.2)
BMI (kg/m^2^)	32.5 (19.0)	32.5 (19.0)	32.5 (19.0)
Waist (cm)	110 (15)	109 (15)	109 (15)
Hip (cm)	111 (14)	111 (14)	112 (14)
W/H ratio	0.99 (0.09)	0.98 (0.08)	0.98 (0.08)

BMI: body-mass index; SD: standard deviation; W/H: waist : hip ratio.

**Table 7:** Patients with ≥1 episode of hypoglycaemia during the 4 weeks preceding baseline and assessments at 3 and 6 months.

Patients, *n*/total (%)	Baseline	Month 3	Month 6
Patients with hypoglycaemia	115/469 (24.5)	66/469 (14.1)^^^	61/469 (13.0)^∗∗^
Severe^∗^			
Day	2/115 (1.7)	0	0
Night	3/115 (2.6)	0	0
Symptomatic^∗^			
Day	69/115 (60.0)	44/66 (66.7)	41/61 (67.2)
Night	76/115 (66.1)	18/66 (27.3)^∗∗^	9/61 (14.8)^∗∗^
Symptomatic confirmed^∗^			
Day	50/115 (43.5)	46/66 (69.7)	50/61 (82.0)
Night	47/115 (40.9)	17/66 (25.8)^^^^	17/61 (27.9)^∗∗∗^

^∗^The total number of patients with hypoglycaemia at each assessment was used as the denominator for calculating the percentages; ^∧^*p* < 0.0001, ^∗∗^*p* < 0.00001, ^∧∧^*p* < 0.0407, and ^∗∗∗^*p* < 0.0880 versus baseline (chi-squared test).

**Table 8:** Number of symptomatic and symptomatic confirmed hypoglycaemia episodes per patient (mean ± SD), among those with ≥1 episode of hypoglycaemia during the 4 weeks preceding baseline and assessments at 3 and at 6 months.

Hypoglycaemia	Baseline	Month 3	Month 6
Symptomatic			
Day	2.5 (1.8)	2.2 (1.3)	2.3 (1.5)
Night	2.3 (2.0)	1.8 (1.0)	1.3 (0.7)^∗^
Symptomatic confirmed			
Day	1.8 (1.0)	1.8 (1.1)	1.8 (1.1)
Night	1.7 (0.9)	1.9 (1.0)	1.4 (0.6)^∗^

SD: standard deviation. ^∗^NS (*t*-test).

## Data Availability

I would like to state that the data supporting conlusions of the study can be accessed on request. To request for data, the readers should contact me directly as the corresponding author.
